# Spontaneous Radiological Disappearance of a Large Staghorn Calculus in an Atrophic Kidney With One Percent Function

**DOI:** 10.7759/cureus.101606

**Published:** 2026-01-15

**Authors:** Mohammad Alabd

**Affiliations:** 1 Urology, United Lincolnshire Teaching Hospitals NHS Trust, Lincolnshire, GBR

**Keywords:** disappearance, kidney, minimal functioning kidney, spontaneous, spontaneous disappearance, staghorn, staghorn calculus, stone

## Abstract

Staghorn calculi are usually managed with active intervention to prevent progressive renal damage, and spontaneous resolution is exceedingly rare. We report the complete radiological resolution of a large staghorn calculus in an atrophic kidney with negligible residual function. An 83-year-old man was found incidentally to have a 4.3 cm right staghorn calculus in an atrophic kidney with only one percent function on mercaptoacetyltriglycine (MAG3) renography. As the patient was asymptomatic, had minimal renal function, and significant comorbidities, conservative management with watchful waiting was chosen. Follow-up imaging one year later demonstrated complete disappearance of the calculus on plain radiography and non-contrast computed tomography, with no evidence of fragmentation, passage, or extrusion, while the contralateral kidney remained unchanged. Although rare, spontaneous resolution of staghorn calculi has been described previously, particularly in poorly functioning kidneys, and potential mechanisms include dissolution of struvite or carbonate-apatite stones in a sterile or treated urinary tract and progressive renal atrophy reducing stone viability. This case adds to the limited evidence suggesting that conservative management may be appropriate in carefully selected patients with staghorn calculi in non-functioning or minimally functioning kidneys, where radiological surveillance may be sufficient and routine active intervention may not always be necessary.

## Introduction

Staghorn calculi are complex renal stones that occupy the renal pelvis and extend into multiple calyces, and are most commonly composed of infection-related minerals such as struvite (magnesium ammonium phosphate) and/or carbonate apatite [1]. These stones are frequently associated with chronic infection by urease-producing organisms and, if left untreated, may lead to progressive renal damage, recurrent infection or sepsis, and eventual loss of renal function [2,3].

Spontaneous radiological disappearance of staghorn calculi is rare and has been reported mainly in isolated case reports. Owda and Turney described complete radiological resolution of a complicated staghorn calculus without surgical intervention in 1995 [2]. In contrast, apparent spontaneous “resolution” more commonly reflects the stone's extrusion rather than true dissolution, for example, via the formation of a nephrocutaneous fistula, as reported by Purkait et al. in 2016 [3].

Standard management of staghorn calculi involves active intervention aimed at complete stone clearance and preservation of renal function [4,5]. Percutaneous nephrolithotomy (PCNL), which involves percutaneous access to the renal collecting system to fragment and remove stone material, is generally considered the preferred first-line approach for most staghorn calculi due to the high stone-free rates and the potential to preserve renal function [6,7]. However, in patients with non-functioning or minimally functioning kidneys - particularly those with advanced age or significant comorbidity - the optimal management strategy is less clearly defined and often requires individualised decision-making [5].

Complete spontaneous radiological disappearance of a staghorn calculus without evidence of migration, fragmentation, or extrusion remains an uncommon clinical observation [2]. We report such a case in an elderly patient in whom a large staghorn calculus was identified in an atrophic kidney with essentially no residual function and was managed conservatively.

## Case presentation

An 83-year-old male patient was incidentally found to have a large right-sided renal calculus during cross-sectional imaging performed for unrelated indications. The initial computed tomography (CT) scan was requested to further characterise a small right renal lesion measuring approximately 1 cm, which was subsequently deemed clinically insignificant. At the time of diagnosis, the patient was asymptomatic, with no history of flank pain, haematuria, lower urinary tract symptoms, recurrent urinary tract infections, or constitutional symptoms. There was no documented prior history of nephrolithiasis or urological intervention. His medical history was notable for multiple comorbidities. Physical examination was unremarkable, with no flank tenderness or signs of systemic infection.

Baseline laboratory investigations demonstrated stable renal function with an estimated glomerular filtration rate (eGFR) of approximately 55 mL/min/1.73 m² and no evidence of systemic inflammation. Urinalysis and urine culture were not available, as there was no clinical suspicion of urinary tract infection at presentation.

**Figure 1:**
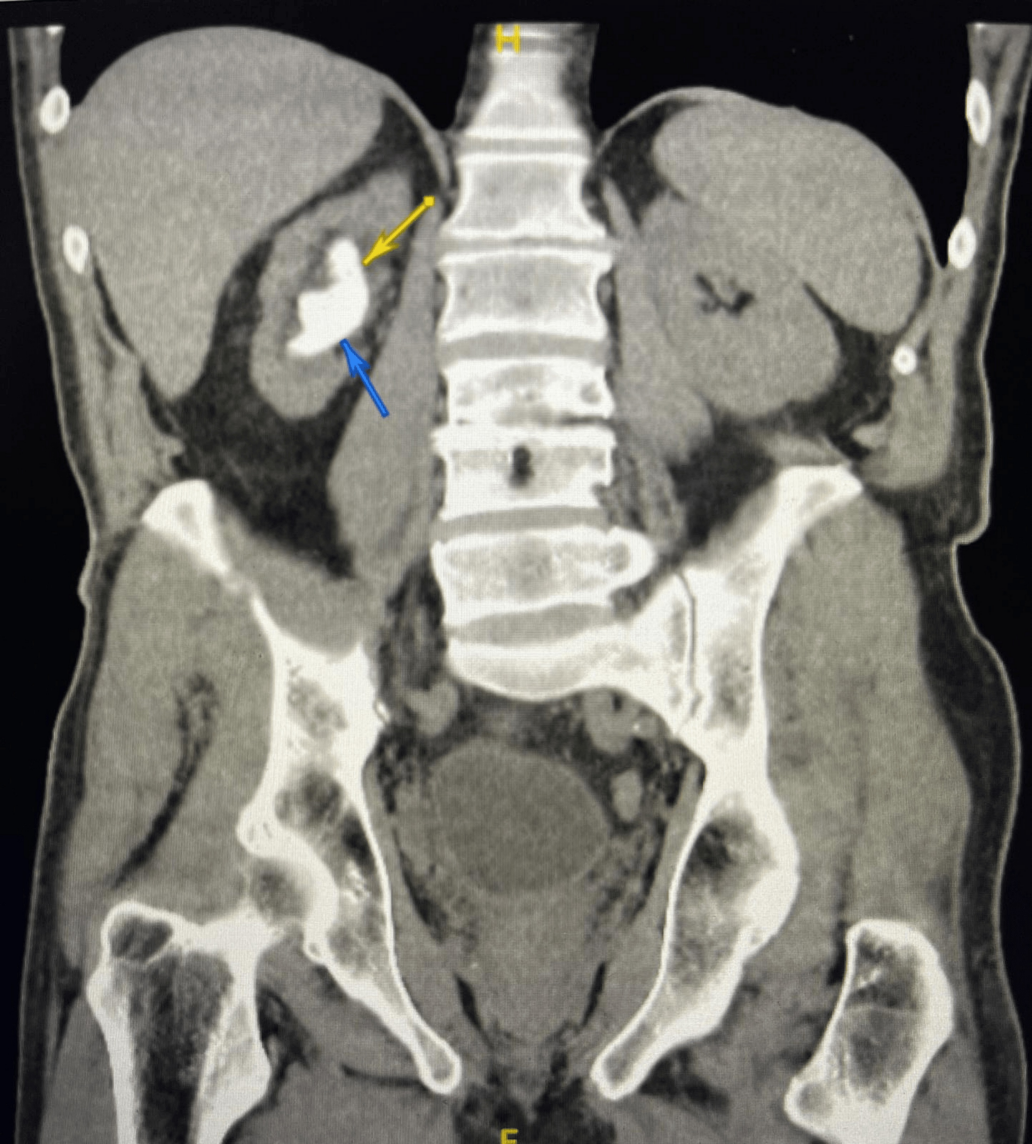
Right staghorn calculus on CT Coronal non-contrast computed tomography scan demonstrating a large right-sided staghorn calculus occupying the renal collecting system (arrows), with associated cortical thinning consistent with renal atrophy.

Initial imaging revealed a large staghorn calculus occupying the right renal pelvis and calyces, measuring approximately 4.3 cm in maximal dimension, with associated marked cortical thinning consistent with an atrophic kidney (Figure 1).

Stone attenuation measured approximately 502 Hounsfield units on non-contrast CT. Mild dilatation of the proximal right ureter was also noted. Given the need to assess both renal function and urinary drainage in this context, functional evaluation was performed using a technetium-99m mercaptoacetyltriglycine (MAG3) renogram rather than a dimercaptosuccinic acid (DMSA) scan. The MAG3 study demonstrated severely reduced right renal function, contributing approximately 1% to the total renal function, with preserved function of the contralateral kidney.

In view of the patient’s absence of symptoms, negligible residual function of the affected kidney, and significant comorbidity burden, management options were discussed in a multidisciplinary setting. Given the high operative risk and limited potential functional benefit of intervention, a decision was made to pursue conservative management with close clinical and radiological surveillance rather than immediate surgical treatment.

**Figure 2:**
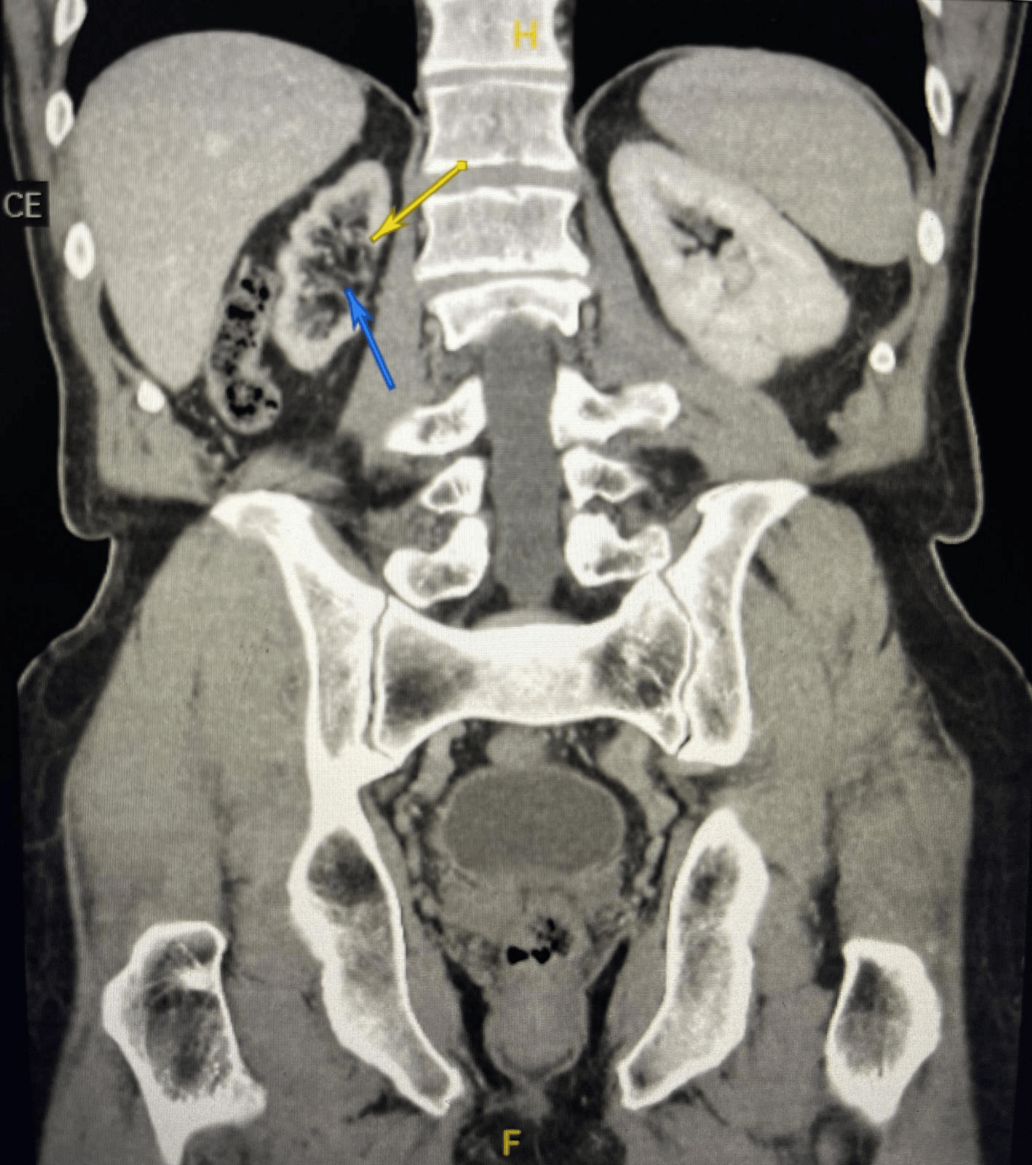
Follow-up CT showing resolution of the staghorn calculus Follow-up contrast-enhanced computed tomography scan obtained approximately one year later demonstrating complete radiological disappearance of the previously identified right staghorn calculus, with persistent right renal atrophy.

Follow-up imaging was performed approximately one year later using renal triple-phase CT. On the non-contrast phase, the patient demonstrated complete radiological disappearance of the previously documented staghorn calculus, with no evidence of residual stone material, fragmentation, migration, or distal ureteric calculi (Figure 2).

The right kidney remained atrophic in appearance, and the contralateral kidney was unchanged. The patient remained clinically well throughout the follow-up period, with no reported urological symptoms or episodes of urinary tract infection.

## Discussion

Rarity and literature context

Spontaneous radiological disappearance of staghorn calculi is exceptionally rare and has been reported almost exclusively in isolated case reports. Owda and Turney described complete radiological resolution of a complicated staghorn calculus without surgical intervention in 1995, representing one of the earliest documented examples of this phenomenon [2]. Since then, only a small number of similar observations have been reported, underscoring the exceptional nature of true spontaneous disappearance.

More commonly, apparent spontaneous resolution of staghorn calculi reflects stone extrusion rather than true dissolution, typically occurring via the formation of a nephrocutaneous fistula. This mechanism has been described, for example, in a paediatric patient reported by Purkait et al. in 2016 [3]. In contrast, the present case demonstrated complete radiological disappearance without evidence of migration, fragmentation, fistula formation, or distal ureteric calculi, making this presentation particularly unusual.

Staghorn calculi are most frequently composed of infection-related stones, with approximately 75% consisting of struvite (magnesium ammonium phosphate) and/or carbonate apatite [1]. These stones are strongly associated with chronic infection by urease-producing organisms and, if left untreated, may result in progressive renal damage, recurrent sepsis, and loss of renal function [1,4]. Consequently, contemporary guidelines advocate active intervention in most cases to achieve complete stone clearance and prevent further morbidity [4-7]. The absence of long-term follow-up data in reported cases, including the present one, limits definitive conclusions regarding subsequent infection risk and long-term renal outcomes.

Potential mechanisms of disappearance

Several mechanisms may plausibly contribute to spontaneous radiological disappearance of a staghorn calculus. Stone composition is likely to play a central role; infection-related calculi composed predominantly of struvite or carbonate apatite may theoretically undergo gradual dissolution under favourable urinary conditions, particularly following suppression or resolution of chronic infection and changes in urinary pH [1,8]. Although pharmacological dissolution strategies, including urease inhibition, have demonstrated reductions in stone burden in selected cases, their role in routine clinical practice remains limited [6].

Severe renal atrophy and markedly reduced renal function may also contribute. A poorly functioning or near-non-functioning kidney produces minimal urine and exhibits reduced metabolic activity, potentially limiting ongoing crystal nucleation and allowing slow litholysis to predominate over time. An alternative explanation is unrecognised micro-fragmentation with silent passage of small stone components; however, the absence of radiological evidence of migration, distal obstruction, or ureteric calculi in this case makes this mechanism less likely [3,4].

Management considerations

Standard management of staghorn calculi involves active intervention aimed at achieving complete stone clearance and preserving renal function. PCNL is widely accepted as the preferred first-line treatment due to its high stone-free rates and potential for renal function preservation [4,6,7]. However, management decisions must be individualised, particularly in elderly or comorbid patients with negligible residual renal function.

In patients with predominantly non-functioning kidneys, minimal or absent symptoms, and high operative risk, the benefits of surgical intervention may be limited. In such highly selected cases, conservative management with close clinical and radiological surveillance may represent a reasonable option [5]. This approach should be interpreted cautiously and should not be extrapolated to younger or symptomatic patients, or to those with meaningful residual renal function, in whom active intervention remains the guideline-recommended standard of care.

## Conclusions

Although active intervention remains the cornerstone of management for staghorn calculi, this case adds to the limited body of evidence demonstrating that complete spontaneous radiological disappearance can occur. In carefully selected patients - particularly elderly individuals with negligible residual renal function, significant comorbidity burden, and minimal symptoms - conservative management with close clinical and radiological surveillance may represent a reasonable alternative to immediate surgical intervention.

Importantly, this approach should be interpreted cautiously and should not be extrapolated to younger or symptomatic patients, or to those with meaningful residual renal function, in whom active intervention remains the standard of care. Further reporting of similar cases with longer-term follow-up is necessary to define patient selection criteria and better understand long-term outcomes.
